# Neurovascular manifestations in patients with COVID-19: a case series

**DOI:** 10.31744/einstein_journal/2022AO6562

**Published:** 2022-03-29

**Authors:** Juliana Cavalcanti de Freitas Reinaux, Karenn Barros Bezerra, Alexandre Sérgio de Araujo Bezerra, Vanessa Garcia Santana, Daniel Lima Souza, Sarah Alcântara Cardoso, Bruna Arrais Dias

**Affiliations:** 1 Hospital Santa Marta Taguatinga DF Brazil Hospital Santa Marta, Taguatinga, DF, Brazil.

**Keywords:** Coronavirus infections, COVID-19, Stroke, Intracranial hemorrhages, Cerebrovascular diseases, Comorbidity, SARS-CoV-2

## Abstract

**Objective:**

To describe cerebrovascular manifestations in patients hospitalized for treatment of severe COVID-19, highlighting the comorbidities observed, and those that may play a relevant role as risk factors for severe outcomes.

**Methods:**

This case series retrospective analyzed, from June to November, 2020, ten patients admitted to the emergency department, with positive nasopharyngeal swab polymerase chain reaction assay for SARS-CoV-2, presenting with neurological symptoms and positive findings at brain imaging studies.

**Results:**

In this sample, the clinical severity of the symptoms varied from mild to critical. Ischemic stroke was observed in four patients, hemorrhagic events occurred in five cases. Three patients evolved with large parenchymal hemorrhage, and one presented petechial bleeding foci. In one case, we observed subarachnoid hemorrhage associated with bilateral hypodensity in both globus pallidus. Typical posterior reversible encephalopathy syndrome findings were observed in one patient on brain computed tomography.

**Conclusion:**

Patients with neurovascular complications related to COVID-19 had positive findings in brain imaging and neurological symptoms. The pathological entities observed drew attention to the neurological risk of patients with SARS-CoV-2 infection, including worse outcomes in individuals whose medical history includes clinical comorbidities, especially hypertension and obesity.

## INTRODUCTION

Since the beginning of the 2019 coronavirus disease (COVID-19) outbreak, in December 2019, the scientific community has been increasingly mobilized to understand its pathophysiology and control the damage caused by the subsequent pandemic.

There is a clear propensity for the severe acute respiratory syndrome 2 (SARS-CoV-2) coronavirus infection to affect the respiratory system. Therefore, early scientific efforts to improve diagnosis and treatment focused on the pulmonary aspects of the disease. However, the knowledge about the potential neurotropic and neuroinvasive effects of coronaviruses has been established, based on studies conducted on previous epidemics caused by other coronaviruses, such as the severe acute respiratory syndrome coronavirus (SARS-CoV), in 2002 , and the Middle East respiratory syndrome (MERS) coronavirus, in 2012.^(1)^ The mechanism is not yet clear, but some penetration routes have been suggested, such as the olfactory epithelium, blood-brain barrier structures, and transsynaptic transmission.^(2)^

In addition, the virus marked affinity for angiotensin-2 converting enzyme (ACE2) receptors makes it potentially aggressive to other organs and systems, including the central nervous system.^(3)^

In severe cases, patients may develop mechanisms by which COVID-19 may increase the risk of events, such as ischemic stroke, large vessel occlusion, intraparenchymal hemorrhage, and central venous thrombosis. These mechanisms include hypercoagulability with elevated levels of D-dimer; cytokine storm syndrome, characterized by the rapid accumulation of T cells and macrophages, which release massive levels of cytokines into the bloodstream, and cardioembolic events due to virus-related cardiac injury.^(4,5)^

Additionally, some comorbidities widely observed among patients with severe COVID-19 are risk factors for stroke. Obesity stands out as a documented risk factor associated with a poor outcome in COVID-19, and a condition that leads to an inherent pro-inflammatory and pro-thrombotic state.^(3)^ Furthermore, in patients with high blood pressure levels, hypertensive encephalopathy may be a cause not always considered for altered mental status and prolonged ventilation times.^(6)^

In the context of the current COVID-19 pandemic, neurological manifestations, especially neurovascular complications, are becoming increasingly important and emerge as a crucial prognostic factor. The pathophysiology of COVID-19 makes these conditions a life-threatening risk, which must be known and expected by the medical team.

## OBJECTIVE

To describe the cerebrovascular manifestations in patients hospitalized for treatment of severe COVID-19, highlighting the comorbidities found that may play a relevant role as risk factors for severe outcomes.

## METHODS

This study was approved by the Ethics and Research Committee (CEP) of *Hospital Santa Marta* (HSM) and is registered under CAAE: 38359120.2.0000.8101, final opinion 4.316.613.

The Informed Consent Form was waived by the Research Ethics Committee of the organization, since this was a non-interventional retrospective study, which only used information from medical records, organizational information systems, and imaging exams already performed, with no alteration or interference in the management of the participants in the research study, and consequently without adding risks or harm to their well-being, with anonymous handling and analysis of the data, without identification of individual participants.

### Study group

Patients admitted to the emergency room from June to November 2020 with a diagnosis of acute SARS-CoV-2 infection confirmed by a positive polymerase chain reaction (PCR) test and with an indication for hospital admission and treatment were retrospectively evaluated.

As inclusion criteria, the emergence of neurological deficits that required brain imaging assessment, with positive findings, was adopted. Twelve patients met these criteria. Patients whose imaging findings resulted from global hypoxic-ischemic injury after cardiorespiratory arrest were excluded from the sample, which occurred in two cases.

The final sample was then composed of ten patients, five females, aged 38 to 68 years (mean age 57.2 years), and five males, aged 37 to 68 years (mean age 55.2 years). Medical comorbidities included hypertension in seven patients, obesity in five, monoclonal gammopathy, cardiac arrhythmia, and diabetes mellitus in one patient each.

The protocol for treatment and support of COVID-19 adopted by the organization was offered to all patients in this study. This protocol included enoxaparin in a prophylactic dose, continuing to a therapeutic dose in cases where D-dimer >1.0 was observed; ceftriaxone and azithromycin; osetalmivir until the H1N1 test result was obtained; dexamethasone 6mg; inhaled bronchodilation with a combination of formoterol and budesonide, in addition to salbutamol associated with ipratropium. Ventilatory support was customized to each case.

### Brain imaging

According to the clinical setting and the findings in previous exams, computed tomography (CT), magnetic resonance imaging (MRI), computed tomography angiography (CTA) and magnetic resonance angiography (MRA) were performed, according to each pathological finding.

Non-contrast head CT was performed as the initial test in all patients. After the first evaluation, MRI was required in four of the cases. In addition, three of the patients underwent angiographic study, two by CTA and one by MRA.

Head CT and CTA were performed in a 64-channel Philips Brilliance CT scanner, 120kV, 300mAs, and 5mm slice thickness.

Before administering the contrast to perform CTA, the saline test was performed with a bolus of approximately 10mL and a flow rate of approximately 5.5mL/s. The calculation basis for the amount of the non-ionic iodinated contrast agent (Optiray^®^ 350) used was approximately 1.3 to 1.5mL/kg.

Computed tomography angiography images were acquired from the aortic arch to the cortical intracranial vessels, automatically performed when the contrast agent reached the ascending aorta, with a flow rate of 4.5mL/s administered by injection pump (Mallinckrodt), followed by a 30mL bolus of saline, with a flow rate of 5.5mL/s.

For MRI and MRA images, a 3.0 Tesla system (Philips 3T) was used. The protocol included three-dimensional T1-weighted (magnetization prepared rapid acquisition with gradient echo) images, three-dimensional fluid-attenuated inversion recovery (3D FLAIR) images, susceptibility-weighted inversion recovery images, T2-weighted diffusion and T1-weighted three-dimensional spin-echo perfusion images using gadolinium. In case of suspected acute stroke, axial FLAIR sequences, diffusion-weighted and susceptibility-weighted images, arterial spin labeling and three-dimensional arterial time of flight sequences were performed.

### Image evaluation

All imaging studies were assessed in consensus by two radiologists with at least 5 years of experience in neuroimaging.

### Case series

#### Patient 1

A 64-year-old hypertensive and obese woman, hospitalized with respiratory symptoms for 14 days, developing headache and syncope in the clinical course. A CTA showed occlusion in the distal portion of the M2 segment of the left middle cerebral artery. The brain MRI showed an area of recent ischemia involving the supramarginal gyrus, left parietal operculum and insula, with a small penumbra area in the relative cerebral blood volume and mean transit time.

#### Patient 2

**Figure 1:**
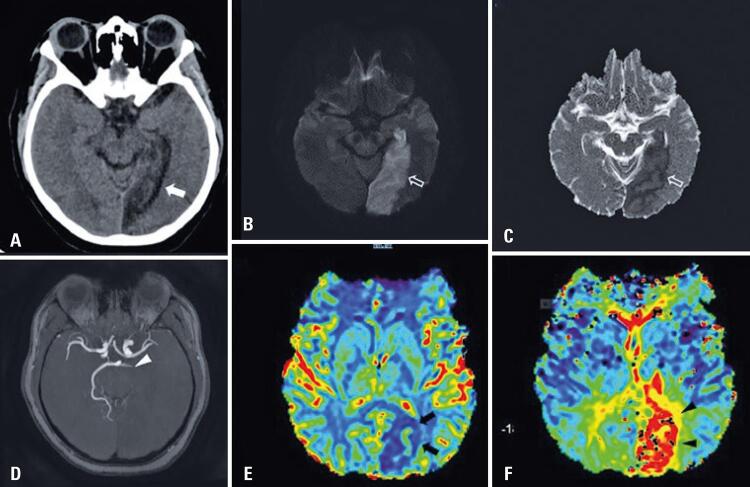
Computed tomography of patient 2. (A) Subacute ischemic lesion involving the left temporal and occipital lobes (white arrow). Magnetic resonance imaging (B and C) showing true restriction, which is seen in the diffusion images and in the apparent diffusion coefficient (empty arrows). Magnetic resonance angiography (D): left posterior cerebral artery occlusion (white arrowhead). Perfusion study (E and F): reduced relative cerebral blood volume (black arrows) and increased mean transit time (black arrowhead) seen in the area corresponding to the ischemic lesion, without significant penumbra

A 38-year-old hypertensive and obese woman, hospitalized with dyspnea. She was having respiratory symptoms for 7 days, and was transferred to the intensive care unit (ICU). Two days later, she developed paresthesia, decreased strength in her right limbs, and uncontrolled hypertension. A head CTA was performed, showing a subacute ischemic lesion involving the left temporal and occipital lobes. Magnetic resonance angiography showed left posterior cerebral artery occlusion, with reduced relative cerebral blood volume and increased mean transit time observed in the area corresponding to the ischemic lesion, without significant penumbra area (Figure 1).

#### Patient 3

A 68-year-old male with no relevant medical history was admitted to hospital after 12 days of self-treatment with ivermectin and azithromycin for COVID-19. On the 21^st^ day of hospitalization, he presented with paresis in the right upper limb, dysarthria and dyslalia. The CT showed an area of ischemia involving the left parietal lobe, and the CTA showed a shortage of vessels from the M3 and M4 segments of the left middle cerebral artery.

#### Patient 4

A 77-year-old hypertensive male with cardiac arrhythmia, in isolation for 7 days, and an episode of syncope with spontaneous improvement 5 days before. He was hospitalized after a new episode of syncope and lowered level of consciousness. A CTA showed thrombosis of the right internal carotid artery immediately after the carotid bulb, extending to the intracranial portion, in addition to a filling defect in the M1 segment of the right middle cerebral artery. There was also a filling defect in the left sigmoid sinus, extending to the ipsilateral internal jugular vein. A follow-up CT performed 2 days later showed extensive hemorrhagic transformation, with a parenchymal hematoma in the right nucleocapsular region, right frontoparietotemporal subarachnoid hemorrhage and diffuse brain edema of the right hemisphere.

#### Patient 5

**Figure 2:**
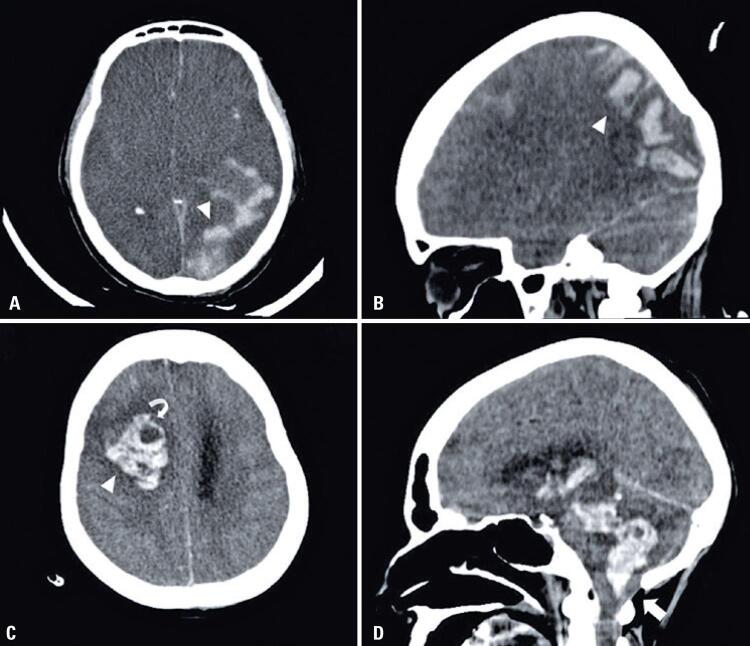
Computed tomography of patients 5 (A) and 6 (B). Diffuse brain edema, loss of gray and white matter differentiation, and large parenchymal hematomas (white arrowhead). Subarachnoid hemorrhage is also present. Computed tomography of patient 7 (C): hemorrhage with heterogeneous attenuation and hypodense areas in between (curved arrow), indicating active bleeding. Reformatted sagittal plane of the patient’s computed tomography scan (D): diffuse edema of the brain parenchyma, with brainstem and cerebellar tonsil herniation, through the foramen magnum (white arrow)

A 48-year-old hypertensive and obese male, was admitted to the hospital with a low fever, cough and anosmia for 4 days. He developed progressive worsening of the respiratory pattern in the clinical course, and was intubated and sedated. After extubation, no satisfactory awakening occurred in the following 24 hours. A head CT was performed 29 days after the onset of symptoms, showing diffuse cerebral edema, in addition to a large parenchymal hematoma on the left. The patient progressed to brain death, confirmed by two clinical examinations and by angiography (Figure 2).

#### Patient 6

A 52-year-old hypertensive and obese woman, hospitalized with cough, fever and anosmia for 3 days. She progressed with worsening of breathing pattern and required mechanical ventilation for 28 days. Thirty-five days after the onset of symptoms, she had a decreased level of consciousness. The CT showed diffuse brain edema, parenchymal hematomas in the right temporal, parietal and occipital lobes, subarachnoid hemorrhage, and subdural hematomas. The patient deteriorated to brain death, confirmed by two clinicians and by transcranial Doppler (Figure 2).

#### Patient 7

A 68-year-old woman, with no reported past medical history, hospitalized with cough and mild chest pain. During hospitalization she developed dyspnea and hypoxemia and was admitted to the ICU. One month later, she developed hypoxemia, hemodynamic shock, and bilateral mydriasis, and tracheal intubation and sedation were performed. A head CT performed 40 days after the onset of symptoms showed intraparenchymal hemorrhage in the right frontal lobe, with contralateral deviation of the midline structures, uncal herniation and herniation of cerebellar tonsils and brainstem, associated with supra- and infratentorial intraventricular hemorrhage (Figure 2). Bilateral subarachnoid hemorrhage and severe diffuse cerebral edema were observed. The patient was entered on a brain death protocol, progressing to refractory shock and death.

#### Patient 8

A 64-year-old hypertensive, diabetic, and obese woman, admitted with cough, myalgia and abdominal pain, and referred to the ICU. Eleven days after admission, she developed hypoxemia, requiring orotracheal intubation, and suffered a cardiopulmonary arrest for 8 minutes. Three days later, a head CT showed small subarachnoid hemorrhages in the left frontal region, and bilateral hypodensities in both globus pallidus. Seventeen days after the onset of symptoms, the patient developed hemodynamic shock and died.

#### Patient 9

**Figure 3:**
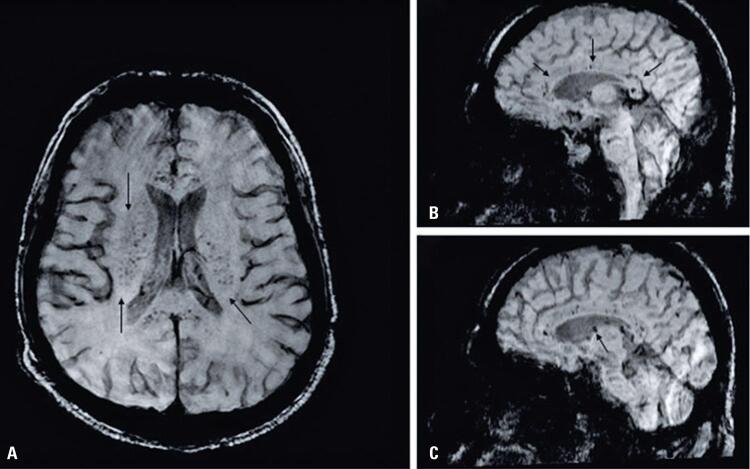
Susceptibility-weighted images in the axial plane of patient 9 (A): multiple petechial hemorrhages in the nucleocapsular regions (black arrows). Susceptibilityweighted images in the sagittal and parasagittal planes (B and C): multiple petechial hemorrhages in the corpus callosum, cingulate gyrus, thalamus, thalamus-brain transition, and pons (black arrow)

A 37-year-old hypertensive man, hospitalized presenting cough for 5 days. After 3 days, he was admitted to the ICU due to dyspnea and low partial pressure of oxygen (PaO_2_) (55.9mmHg). Forty-five days after onset of symptoms, he developed bilateral lower limb paresthesia, with normal head CT. The MRI showed multiple petechial hemorrhages in the corpus callosum, cingulate gyrus, nucleocapsular regions, thalamus, pons, and some sparse lesions in the cerebral hemispheres (Figure 3), probably related to a coagulopathy associated with COVID-19.

#### Patient 10

**Figure 4:**
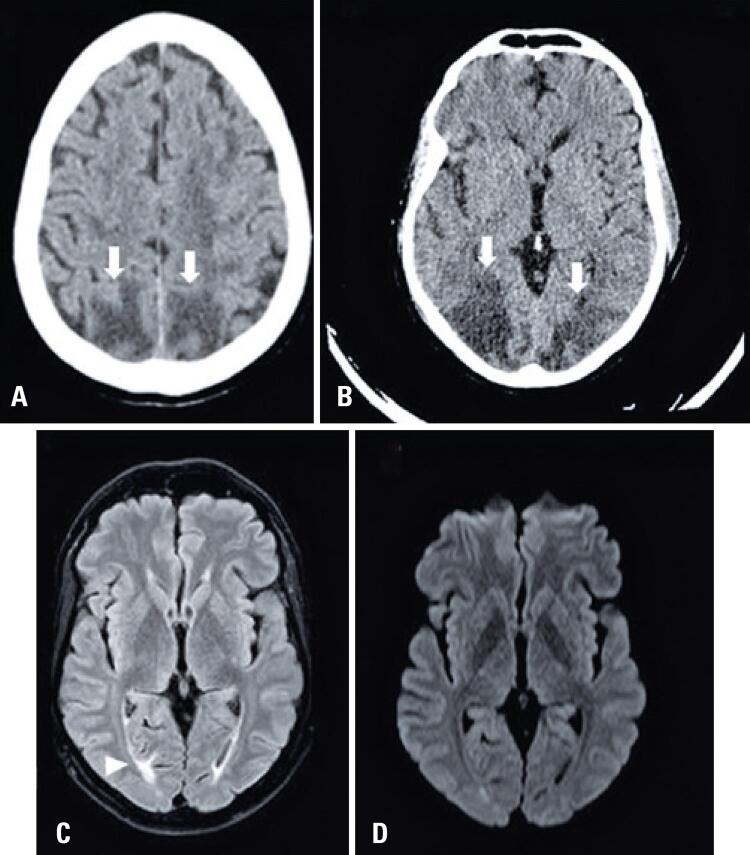
Patient 10. Computed tomography scan. (A and B) Axial sections show hypodensity in the subcortical white matter of the parietal and occipital lobes (white arrows). (C) Axial fluid-attenuated inversion recovery images: hyperintense area in the left parieto-occipital white matter (white arrowhead), with reduced dimensions in comparison to the lesion area seen on the previous computed tomography scan. Diffusion-weighted (D) images in the axial plane. There were no diffusion restriction areas

A 46-year-old male, with a clinical history of monoclonal gammopathy, hospitalized with fever, myalgia, dyspnea, headache, retro-orbital pain and nausea. During hospitalization, there was a worsening of the breathing pattern, requiring orotracheal intubation. Weaning from sedation was started 35 days after the onset of symptoms, but the patient had an unsatisfactory awakening. A head CT showed parieto-occipital and frontal edema, predominantly affecting the white matter, in addition to frontal subarachnoid hemorrhage. The patient developed no deficits, and the brain MRI performed 30 days after the first exam showed a significant improvement from the previous findings (Figure 4).

## RESULTS

In this sample, the clinical presentation ranged from mild symptoms, such as headache and paresthesia, to severe conditions, with decreased level of consciousness and death. Patients had a wide range of neurological syndromes, including ischemic stroke and hemorrhagic events (Table 1), with the onset of manifestations ranging from 7 to 45 days (mean 24.6 days) after the onset of symptoms of COVID-19.

**Table 1:** Significant findings in the imaging exams performed

Image characteristics	n*
Ischemic stroke	4
Stroke characteristics	
Single territory	4
Multiple territories	0
Large vessels involved	2
Concomitant venous thromboembolism	1
Occurrence during therapeutic anticoagulation	4
High levels of D-dimer	4
PRES	1
Bilateral basal ganglia involvement	1
Intraparenchymal hemorrhage	3
Petechial microbleeds	1

* Total number of patients with neurovascular manifestations documented by brain imaging in patients with COVID-19.

PRES: Posterior reversible encephalopathy syndrome.

Ischemic stroke was observed in four patients, and two of them involved a large vessel, such as the internal carotid artery and proximal segments of the posterior and middle cerebral arteries. An MRI perfusion study was performed in two cases, confirming the stroke nucleus in diffusion-weighted imaging (DWI) sequences, with no relevant penumbra area. Hypertension was reported in three of these patients, in two cases associated with obesity and, in one case, with cardiac arrhythmia. In one case there was no known pathological history.

Bleeding events occurred in five cases. Three patients developed massive parenchymal and intraventricular hemorrhage. Among them, two patients were hypertensive and obese, and one patient had no known comorbidities. One hypertensive patient, with a previous head CT scan with no significant findings, presented with sparse petechial bleeding foci observed only on susceptibility weighted imaging (SWI). In one case, a subarachnoid hemorrhage associated with bilateral hypodensities in the globus pallidus was observed. In this case, there was also a clinical history of hypertension, obesity, and diabetes.

Typical posterior reversible encephalopathy syndrome (PRES) findings were observed on CT in one patient with a history of hypertension. A subsequent MRI showed a significant improvement in the findings, without significant sequelae.

At the end of the analysis, the combination of hypertension and diabetes was found in five patients, occurring in 62.5% of severe outcomes (ischemic stroke and hemorrhage) and in 60% of cases that progressed to death.

## DISCUSSION

This case series aimed to draw attention to possible neuroradiological manifestations in patients with COVID-19, with an emphasis on the broad spectrum of neurovascular complications, such as ischemic stroke, hemorrhagic stroke, micro-hemorrhages, and venous sinus thrombosis.^(7)^

Although the exact prevalence has been difficult to establish, ischemic stroke emerges as a frequent cerebrovascular complication in the COVID-19 scenario.^(8)^ Some authors suggest that, in these patients, this entity may have distinct characteristics, including occlusion of large vessels, involvement of multivessel territories, occurrence despite therapeutic anticoagulation, concomitant venous thromboembolism, very high levels of D-dimer, and evidence that ischemic stroke usually occurs later than the onset of symptoms.^(9)^ In this cohort, the events occurred during anticoagulant therapy, and the other characteristics described herein could be observed, to a greater or lesser degree - except for the involvement of multiple territories.

As observed in this study, bleeding events are associated with worse outcomes, and they are less reported in the literature.^(3)^ Apparently, extensive hemorrhages may result from the hemorrhagic transformation of an ischemic infarction, or may be a complication of venous thrombosis,^(3)^ whereas petechial bleedings observed in SWI are due to diffuse endothelial dysfunction and may be similar to necrotizing encephalopathy.^(10)^

Similar to the pattern observed in studies on SARS-CoV-1 infection, SARS-CoV-2 may incite non-inflammatory encephalopathy, which is characterized by areas of focal restriction and diffuse signal changes on FLAIR MRI sequence, as well as hypodensities basal ganglia on tomography^(10)^ – this last aspect was also observed in a patient in this study.

Classically associated with systemic hypertension and hypertensive encephalopathy, PRES has a controversial pathophysiology, originally attributed to loss of autoregulation or ischemia induced by vasospasm. More recent studies suggest a more comprehensive pathophysiological mechanism, with endothelial dysfunction, hemodynamic stress (hypertensive crisis) and release of cytokines capable of activating endothelial cells — all these factors combined have a key role in increasing vascular permeability.^(11)^ Even relatively moderate fluctuations in pressure may be associated with PRES in patients infected with SARS-CoV-2, perhaps due to the aforementioned endothelial damage and cytokine storm, which characterize the pathogenesis of COVID-19, requiring strict control of blood pressure in these patients.^(6)^

In the scenario of SARS-CoV-2 infection, there is an increased neurovascular risk, probably multifactorial, which can be explained by a combination of a cytokine release syndrome, leading to endothelial dysfunction, hypercoagulability state, cardiopulmonary dysfunction with hypoxia, and hemodynamic failure accentuated by the breakdown of the blood-brain barrier mediated by viral invasion.^(3,8)^

A limitation of this study is the small number of cases, which may not include other neurological alterations related to COVID-19, in addition to the fact that the study, due to its nature as a case series, is retrospective.

## CONCLUSION

Patients hospitalized with SARS-CoV-2 infection had neurovascular complications, with positive brain imaging findings, and mild to critical neurological symptoms. Clinical comorbidities were frequently found, especially hypertension and obesity, and they were associated with worse outcomes and death. The pathological entities observed were ischemic stroke, intraparenchymal hemorrhage, microbleeds, subarachnoid hemorrhage, dural sinus thrombosis, and posterior reversible encephalopathy syndrome, with worse prognosis in individuals whose clinical history included known pro-inflammatory and pro-thrombotic entities, since there seems to be an overlap between them and the set of factors that trigger endothelial damage and a state of hypercoagulability, which characterize COVID-19.
